# Brown Algae as a Valuable Substrate for the Cost-Effective Production of Poly-γ-Glutamic Acid for Applications in Cream Formulations

**DOI:** 10.3390/polym16142091

**Published:** 2024-07-22

**Authors:** Mattia Parati, Catherine Philip, Sarah L. Allinson, Barbara Mendrek, Ibrahim Khalil, Fideline Tchuenbou-Magaia, Marek Kowalczuk, Grazyna Adamus, Iza Radecka

**Affiliations:** 1 Faculty of Science and Engineering, University of Wolverhampton, Wolverhampton WV1 1 LY, UKibrahim.khalil2@wlv.ac.uk (I.K.); f.tchuenbou-magaia@wlv.ac.uk (F.T.-M.); 2 Research Institute in Healthcare Science, Faculty of Science and Engineering, University of Wolverhampton, Wulfruna Street, Wolverhampton WV1 1LY, UK; 3 Biomedical and Life Sciences, Lancaster University, Furness Building, Lancaster LA1 4YG, UK; s.allinson@lancaster.ac.uk; 4 Centre of Polymer and Carbon Materials, Polish Academy of Sciences, M. Curie-Sklodowskiej 34, 41-819 Zabrze, Poland; bmendrek@cmpw-pan.pl (B.M.); gadamus@cmpw-pan.pl (G.A.); mkowalczuk@cmpw-pan.pl (M.K.)

**Keywords:** γ-PGA, cream formulations, biotechnology, *Bacillus*, hydration, delivery systems, skin microbiota

## Abstract

Poly-γ-glutamic acid (γ-PGA) is a carboxylic-acid-rich, bio-derived, water-soluble, edible, hydrating, non-immunogenic polymer produced naturally by several microorganisms. Here, we re-emphasise the ability of *Bacillus subtilis* natto to naturally produce γ-PGA on whole seaweed, as well as for the yields and chemical properties of the material to be affected by the presence of Mn^(2+)^. Hyaluronic acid (HA) is an extracellular glycosaminoglycan which presents a high concentration of carboxylic acid and hydroxyl groups, being key in fulfilling numerous applications. Currently, there are strong environmental (solvent use), social (non-vegan extraction), and economic factors pushing for the biosynthesis of this material through prokaryotic microorganisms, which is not yet scalable or sustainable. Our study aimed to investigate an innovative raw material which can combine both superior hygroscopicity and UV protection to the cosmetic industry. Comparable hydration effect of commercially available γ-PGA to conventional moisturising agents (HA and glycerol) was observed; however, greater hydration capacity was observed from seaweed-derived γ-PGA. Herewith, successful incorporation of seaweed-derived γ-PGA (0.2–2 *w*/*v*%) was achieved for several model cream systems with absorbances reported at 300 and 400 nm. All γ-PGA-based creams displayed shear thinning behaviour as the viscosity decreased, following increasing shear rates. Although the use of commercial γ-PGA within creams did not suggest a significant effect in rheological behaviour, this was confirmed to be a result of the similar molecular weight. Seaweed-derived γ-PGA cream systems did not display any negative effect on model HaCaT keratinocytes by means of in vitro MTT analysis.

## 1. Introduction

Hyaluronic acid (HA) is a glycosaminoglycan that is found in the extracellular matrix of soft connective tissues [1]. Thanks to its high concentration of carboxylic acid and hydroxyl groups, this material possesses a wide range of applications. High-molecular-weight HA (>10 kDa) has been shown to have wide use in ophthalmology, orthopaedics, cosmetics, and tissue engineering [2,3,4], whereas low-molecular-weight HA (<5 kDa) has been shown to be a versatile backbone for producing complexes that promote angiogenesis, inhibit tumour progression, or induce expression of pro-inflammatory mediators [4]. In particular, HA has been considered of interest for the cosmetic sector as it possesses high water binding capacity [1]. Conventionally, HA is extracted from animal tissue through a technically challenging process which involves grinding, acid treatment, and repeated extraction with organic solvents [4]. Currently, there is a strong environmental (solvent use), social (non-vegan extraction), and economical (HA moisturisers will be valued at USD 7.36 billion by 2033 [5]) drive towards synthesising this biopolymer through microorganisms due to the vegan nature of the resulting material. It has been shown that *Streptococcous* sp., amongst others, naturally produce HA extracellularly for adherence and protection but also as a molecular imitation for evasion of the host immune system during its infection process [4]. However, *Streptococcus* sp. is still widely considered a pathogen capable of producing several endotoxins; to this end, either enzymatic (cell-free) systems are employed (limited to 2000 kDa) [6], or the biochemical pathway for HA synthesis is engineered within non-endotoxin-producing, generally regarded as safe (GRAS) organisms (*Bacillus* sp.) [4,7]. Nonetheless, this approach still sees high variability in molecular weights obtained for HA, relatively low yields at industrial scale (6–7 g/L) due to the high viscosity [8], public perception of genetically modified organism (GMO) products, and very high CAPEX facilities (to handle GMOs) [4]. Further, further broadening the application of HA is the ubiquitous nature of natural hyaluronidases, capable of efficiently depolymerising the polymer and biochemically utilising it as a natural carbon source [9].

Similarly to *Streptococcus* sp., other natural organisms have been shown to produce extracellular material for adherence, protection, and immune evasion. Historically, anthrax from *Bacillus antheracis* was notoriously difficult to identify and treat due to native biosynthesis of poly-D-γ-glutamic acid, an immune-invisible pseudopolypeptide. Similarly to HA, poly-γ-glutamic acid is rich in water-interacting groups (carboxylic acid) and is produced between 10 and 10,000 kDa. 

Numerous prokaryotic and eukaryotic microorganisms have been found to naturally synthesise poly-D/L-γ-glutamic acid (γ-PGA), a biodegradable, water-soluble, non-immunogenic biomacromolecule [10,11,12]. γ-PGA is an edible biopolymer widely consumed in the Asian continent and has been used for several applications, ranging from bioremediation [13,14,15] to tuneable drug delivery systems [16,17,18,19,20]. Such suitability for different applications has been achieved due to the chemico-physical composition of the material, which can be obtained by tuning the conditions of biosynthesis [10,11,21,22,23,24]. In fact, as γ-PGA is synthesised by a transmembrane enzymatic complex instead of a ribosomal complex, both the molecular weight and the D/L-glutamic acid ratio can be varied. It has been previously suggested that both the molecular weight and the D/L-glutamic acid ratio can significantly impact the rheological behaviour of γ-PGA, the water absorption capabilities, and the water holding capacity [25]. Differently to HA, γ-PGA can persist longer in natural environments due to the γ-peptide bond, which is not degradable by means of commonly produced α-proteases [21,24]. Further, being naturally produced by members of the *Bacillus* sp., there is no need to transfer the genetic capacity to other organisms as it has highly developed biosynthetic capacity and capability to grow in industrial fermenters [4]. These factors have been suggested to be crucial to substitute conventional moisturisers [1,4]. Further, γ-PGA has been suggested to possess other beneficial properties, including UV protection [26] and pre-biotic capacity [27], both highly desirable for the next generation of cosmetic solutions.

Similarly to GMO-mediated HA synthesis, the biosynthesis of γ-PGA is predominantly achieved by means of a defined media [12,24,28]. With an outlook on industrial commercialisation and up-scaling, several more circular, complex waste media have been assessed and shown to be suitable for γ-PGA biosynthesis. For instance, ref. [29] employed rotten tomatoes for the biosynthesis of γ-PGA, ref. [24] successfully synthetised γ-PGA from supplemented macroalgae, and ref. [30] employed soybean meal for the biosynthesis of γ-PGA. The use of complex waste media as a substrate for γ-PGA biosynthesis has been further described in [12,31,32].

Here, we aim to demonstrate the ability to effectively alter the chemico-physical properties of γ-PGA through variation in substrate composition; compare the rheological behaviour of γ-PGA creams with commercial creams/lotions; and compare water absorption capacity of γ-PGA, HA, and glycerol. Within this context, seaweed-derived γ-PGA creams are assessed for their hydration, UV protection, and overall biocompatibility against HA, the golden standard in the industry. The aim of this work is to provide an innovative raw material which can combine both superior hygroscopicity and UV protection to the cosmetic industry. The next generation of cosmetic ingredients must be synthesised and isolated in a greener manner, and they need to be vegan friendly, biocompatible, and have comparable or improved properties to HA, the current gold standard in the cosmetic industry.

## 2. Materials and Methods

### 2.1. Biosynthesis of γ-PGA

#### 2.1.1. Microorganism

*Bacillus subtilis* natto (ATCC15245, Manassas, VA, USA) was obtained from the National Collection of Industrial and Marine Bacteria (NCIMB, Aberdeen, UK). Freeze-dried cultures were resuscitated on Tryptone Soya Agar (TSA) (Lab M, Heywood, UK) plates. Next, only highly mucoid colonies were transferred into in shake flasks containing 100 mL of TSB medium (Lab M, Heywood, UK) at 37 °C for 24 h, as described in our previous paper [24].

#### 2.1.2. Fermentation Media

GS Medium Components (Table 1): l-glutamate was purchased from Fischer Chemicals Ltd. (Loughborough, UK), and sucrose, KH_2_PO_4_, Na_2_HPO_4_, NaCl, MgSO_4_·7H_2_O, and Murashige–Skoog vitamin solution were all purchased from Sigma-Aldrich (Irvine, UK). The pH of this medium was adjusted to 6.8 using NaOH purchased from Fischer Chemicals Ltd. (Loughborough, UK).

Macroalgal medium components: *Ascophyllum nodosum* powder (Pure Sea Ltd., Oban, Scotland, UK), l-glutamate (Fischer Chemicals Ltd., Loughborough, UK), and 25 g/L sucrose (Sigma-Aldrich, Irvine, UK).

All standard media were prepared using deionised water and sterilised by autoclaving at 121 °C for 10 min. All complex media were prepared using deionised water and sterilised by autoclaving at 115 °C for 15 min. The sucrose and vitamin solution were filter sterilised separately (0.22 µm, Fischer Chemicals Ltd., Loughborough, UK) and added separately to the medium.

#### 2.1.3. Fermentation Parameters

Batch cultures were carried out as previously described by Parati et al. (2023) [24]. Briefly, bacterial batch cultivation was carried out in 250 mL conical flasks for the 96 h at 37 °C, and agitation was set 150 rpm. Bacterial growth was monitored by viable count [33] and expressed as Log colony-forming units/mL (Log CFU/mL). All results were statistically analysed using Microsoft Excel and GraphPad Prism.

### 2.2. Isolation and Purification of γ-PGA

The material was isolated using methodology described in our previous paper [24]. Briefly, the culture broth was removed from the fermenter and centrifuged at 17,000× *g* for 30 min using a ERMLE Z 300K centrifuge (Wehingen, Germany). The obtained supernatant was poured into 3 volumes of 70% cold ethanol and left overnight at 5 °C to precipitate. Next, the precipitate was collected by filtration using a 0.22 µm paper filter (Fischer Chemicals Ltd., Loughborough, UK). Resulted precipitate was subsequently lyophilised (Alpha 1–4 LSC plus Christ Freeze Dryer, SciQuip Ltd., Bomere Heath, UK). Dry powder was stored in a desiccator at room temperature until further use. Further purification was achieved by using a cross-flow system purchased from Repligen, US, with a 20 cm MidiKros column of 30 KDa cut off from Repligen.

### 2.3. Statistical Analysis

All data were carried out in triplicate and statistically analysed by means of standard deviation, standard error, and two-way ANOVA using Graphpad prism 25.

### 2.4. Characterisation of γ-PGA

The average molecular weight and molecular weight distributions were assessed by gel permeation chromatography (GPC) with a differential refractive index detector (Δn-2010 RI WGE Dr. Bures, Berlin, Germany) and a multiangle laser light scattering detector (DAWN EOS, Wyatt Technologies, Santa Barbara, CA, USA), as described previously [24].

X-ray diffraction (XRD) was quantified as described previously [24]. XRD data were confirmed by means of thermogravimetric and differential scanning microscopy, as described by our team before [24].

The rheology of all cream formulations was analysed using a Malvern Kinexus rheometer to study the viscosity and shear stress rate. The serrated geometries, PU40XSW1383SS and PLS40XS290SS (Malvern Panalytical, UK), were utilised to measure the rheology parameters. The resulting data were further assessed with the aid of the power law model, presented within Equation (1), which describes the power law model, where *k* is the consistency, *n* is the power law index, and *σ* is the shear rate:

(1) $$\sigma ={k(y}^{n})$$

The obtained experimental data were processed with the software rSpace (v 2.1). All samples were analysed in triplicate. Average data were employed to plot viscosity vs. shear rate curves. Standard cream formulations were purchased from The Body Shop (West Sussex, UK) and were used as reference.

### 2.5. Hydration Experiments of γ-PGA

Evaluation of the moisture absorption by γ-PGA was adapted from the protocol described by [34]. In brief, γ-PGA synthesised by microorganisms was lyophilised at the end of the isolation step and subsequently stored in a dehydrator. Before use, all powders were placed at 70 °C for 48 h. Subsequently, the material was placed in a two-beaker system to create a high humidity environment. Within this system, the powder was placed in an inner beaker and subsequently in a bigger beaker filled with 50 mL of distilled water. The two beakers were sealed tightly to prevent any moisture escaping. The samples were maintained at 70 °C for 4 h. During the experiment, the percentage change in γ-PGA was quantified over time at regular hourly intervals. Glycerol and commercial hyaluronic acid were used as controls. The hygroscopicity of the γ-PGA was calculated by means of Equation (2), which quantifies the difference in weight of *W*_1_ the γ-PGA (+H_2_O) and the inner beaker subtracted by *W*_0_, which refers to the weight of the inner beaker only.

(2) $$Hygroscopicity\left(\%\right)=\frac{\left[W_{1}-W_{0}\right]}{W_{0}}*100$$

The hygroscopicity data were compared with existing commercial material as well as known hygroscopic compounds. These have been summarised within Table 2.

### 2.6. Biocompatibility of γ-PGA

Low passage number human HaCaT keratinocytes grown in DMEM supplemented with 10% foetal bovine serum, penicillin (10 units/mL), and streptomycin (10 µg/mL) were trypsinised and plated in a 96-well plate at a density of 1500 cells/well. The cells were then incubated overnight (37 °C, 5% CO_2_). Autoclaved γ-PGA samples were dissolved at a concentration of 2% in supplemented DMEM before double dilution to produce a range of concentrations. It was observed that at concentrations above 2% the γ-PGA caused a decrease in pH, leading to a change in colour of phenol red, and so concentrations above this were not tested. The γ-PGA samples at the indicated concentrations were then added to wells in triplicate. After 72 h, the medium was carefully removed from each well and replaced with medium containing TACS XTT Cell Proliferation reagent (R&D Systems) at the manufacturer’s recommended concentration. The plate was returned to the incubator for 2 h before the absorbance of each well was read. Absorbance values for each condition were averaged and corrected by subtracting the absorbance of a no-cell control. The percentage viability relative to an untreated control was then calculated for each condition. Three independent biological replicate experiments were carried out.

### 2.7. Analytical Evaluation of γ-PGA UV Protection

UV protection was assessed by means of specrophotometric absorbance. All material was diluted to the desired concentration (5%, 0.5%, 0.2%) and subsequently scanned through the UV absorbance spectrum between 200 and 900 nm by means of an Ultrospec 2100 pro (Amersham Biosciences, Cytiva, UK).

### 2.8. Photosensitisation Test

Low-passage-number human HaCaT keratinocytes grown in DMEM supplemented with 10% foetal bovine serum, penicillin (10 units/mL), and streptomycin (10 µg/mL) were trypsinised, washed with antibiotic and phenol red-free DMEM (PSPR-free DMEM), and then suspended at a concentration of 500 cells/mL in PSPR-free DMEM. This cell suspension was then added to 6-well plates at 1 mL/well. The plates were placed in the incubator (37 °C, 5% CO_2_) for 3 h to allow cells to attach. An equal volume of autoclaved commercial γ-PGA (YR Spec) dissolved in PSPR-free DMEM at a concentration of 2% was then added to three wells on each plate. Plates were then either left unirradiated or irradiated with either UVA or UVB for the indicated doses. The UVA source consisted of an array of Philips TLR 36 W “blacklight” tubes. Wavelengths below 320 nm were filtered out using polyester film (No. 130 clear; Lee Filters, UK, spectrally equivalent to Mylar). The spectral irradiance ranged from approximately 330 to 400 nm with a peak output at 365 nm and an intensity of around 140 Wm^−2^. The UVB source consisted of an array of Phillips T140 UVB tubes, emitting a broad spectral irradiance ranging from 275 to 380 nm with peak output at 315 nm and an intensity of 14.7 Wm^−2^. Output spectra and intensities were measured using an IDR150 double monochromator spectroradiometer (Bentham Instruments Ltd., Reading, UK). The plates were then returned to the incubator and incubated for 9 days. Colonies were fixed by removal of the medium followed by addition of 70% ethanol for 20 min. Colonies were stained with GIEMSA and then manually counted. The percentage survival was then calculated based on the average number of colonies in each well for each condition (i.e., +/− γ-PGA) on the irradiated plates relative to the average number of colonies in each well for the equivalent conditions on the unirradiated plate. Three independent biological replicate experiments were carried out.

### 2.9. Rheological Analysis

The rheological testing of all cream formulations (Table 3) was performed via viscometry using a Malvern Kinexus rheometer (Malvern Instrument Ltd., Worcestershire, UK) under the control strain mode. Stainless steel serrated plate geometries (PLS40XS290SS and PU40XSW1383SS (PLS40X)) with 1 mm gap width were used to prevent wall slip and to measure the changing in viscosity as function of the shear rate. The shear rate range was 0.1–100 s^−1^ with a logarithmic variation. After sample loading, a holding period of 5 min was used to allow the sample to recover and to reach the desired temperature, 37 °C. The data obtained from the experimental procedure were processed by the rSpace software. The samples were analysed in triplicate, and the averages of the data points obtained were utilised to plot the curves for viscosity vs. shear rate. Standard cream formulations obtained from the cosmetic company The Body Shop were used as a reference. The curves obtained from the reference samples were compared to that of the cream formulations for analysis.

### 2.10. γ-PGA-Based Cream Formulations

γ-PGA-based cream formulations were prepared as presented in Table 4. Aqueous and oil phase were heated to 50 °C and stirred at 100 rpm to ensure emulsification. The texture as well as feel, visual appearance, and post-application sensation were evaluated, and the final pH of formulations are summarised within Table 5.

### 2.11. Optical Transmittance Spectra of Creams

Cream formulation was spread on a 5 cm square fused quartz plate (UQG Optics Ltd., Cambridge, UK) at 2.5 mg/cm^2^, and an identical plate was placed on top. The transmittance spectrum was then measured on an IDR150 double monochromator spectroradiometer (Bentham Instruments Ltd., Reading, UK) using a CL6 150 W quartz halogen light source. Transmittance at each wavelength was calculated relative to that through the two quartz plates in the absence of cream.

## 3. Results and Discussion

**Figure 1:**
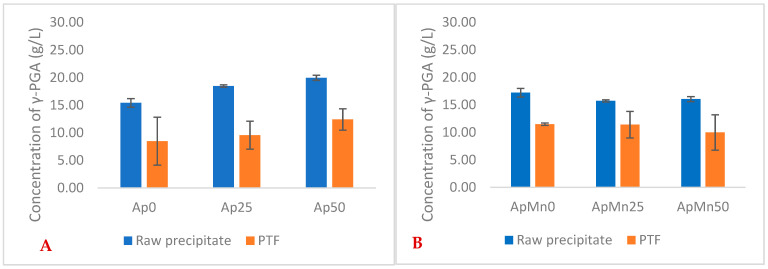
Average yields of γ-PGA (raw and post-tangential flow purification) obtained from Ascophyllum nodosum cultivation media at varying NaCl concentrations (**A**) and varying NaCl + manganese sulphate (**B**). Error bars indicate standard deviation (*n* = 3 shake flask).

Similarly to the defined GS media, the greatest yields with seaweed-based media were observed for 25 g/L NaCl conditions (Figure 1). Differently, higher yields of γ-PGA were obtained with 0 g/L NaCl concentration when in the presence of manganese sulphate. Overall, there was no significant difference in yields between manganese sulphate supplemented and non-supplemented samples in raw precipitates (Figure 1) (*p* = 0.574). Overall, our data suggest that manganese-supplemented substrates provided a higher material yield after tangential flow filtration compared to non-manganese-supplemented substrates.

It has been widely reported that the molecular weight, the crystallinity, and the ion profile of γ-PGA are crucial factors impacting overall material viscosity [35,36]. These parameters have been benchmarked against commercially available γ-PGA samples, which are specifically sold for cosmetic application. The properties of such commercially available polymers were compared to those previously obtained with *Bacillus subtilis* natto cultivated on standard GS media, and algal-media-derived γ-PGA was assessed by means of gel permeation chromatography. Successful biosynthesis of γ-PGA by means of standard GS and seaweed-based media (*Laminaria digitata*, *Saccharina latissimi*, and *Alaria esculenta*) was previously confirmed by means of FT-IR [24]. Analogous procedure has been employed to confirm successful biosynthesis of Ap and ApMn samples reported within Table 6. The physical properties of γ-PGA produced has been summarised within Table 6.

From Table 6, it can be observed that there was an inversely proportional behaviour in both samples with NaCl supplementation and in samples with NaCl + MnSO_4_ supplementation for *Ascophyllum nodosum*-derived substrate, meaning that, with increasing concentrations in NaCl, the molecular weight of γ-PGA synthesised was decreased (see Table 6). Such correlation was also previously reported by [37], but it was not upheld from previous investigations reported by [24], for which 50 g/L NaCl yielded higher-molecular-weight γ-PGA compared to 25 g/L NaCl concentrations (see Table 6). Such variation in NaCl dose–response can significantly be affected by the type of microorganism amongst other possible factors [38]. 

Similarly to standard GS media, with an increase in concentration of NaCl, there was no significant decrease in the average molecular weight of γ-PGA (in both raw and PTF samples) [24] (Figure 1). Such a trend does appear to be upheld with *Ascophyllum nodosum*-based substrates. This suggests that the type of substrate might have a similar NaCl effect on molecular weight. However, differently from what was reported in the literature, 50 g/L of NaCl did not seem to impact crystallinity of the γ-PGA formed. This lack of crystallinity could be due to the low molecular weight of the γ-PGA produced by *Bacillus subtilis* natto in this case compared to the molecular weight of γ-PGA produced in standard GS media [12]. We previously reported that with increases in concentration of NaCl, there is the formation of crystalline material (same microorganisms, same process conditions) [24]. This variation in properties can be directly correlated to the lower molecular weight of the γ-PGA produced through *Ascophyllum nodosum*-based media at 50 g/L NaCl (2,700,000 Da GS compared to 102,000 Da from supplemented *Ascophyllum nodosum* media).

Interestingly, in standard GS media, when supplemented with MnSO_4_, it appears that the concentration of NaCl required to synthesise a crystalline polymer was decreased from 50 g/L to 25 g/L NaCl. This can be explained by the increased polymerisation of D-glutamic acid monomers (as suggested in literature), which results in the overall split between D- and L-glutamic acid monomers closer to 50:50 and ultimately in lower entropy for self-arrangement of γ-PGA chains in a crystalline lattice.

Differently from *Ascophyllum nodosum*-based media, with standard GS media, there is not such a variation between the concentration of NaCl and the molecular weight of the material. Interestingly, in GS media, when there was a supplementation of the media with 0.415 g/L MnSO_4_, there was roughly a halving in the molecular weight of the resulting γ-PGA. This result is in line with data previously reported by [38]. Differently, in *Ascophyllum nodosum*-based media, the supplementation of 0.415 g/L MnSO_4_ resulted in almost a quadrupling of the molecular weight for the 0 g/L NaCl set of experiments, with comparable molecular weights at 50 g/L NaCl concentrations (Table 2). Interestingly, across all *Ascophyllum nodosum* conditions, the resulting γ-PGA presented amorphous behaviour. Such a lack of semi-crystallinity can be expected with such low-molecular-weight polymers; therefore, the effectiveness of NaCl to catalyse higher crystallinity cannot be commented on with this set of experiments. 

In the literature, it has been described that, depending upon the overall ionic prevalence of γ-PGA, its physical arrangement can vary significantly, and ultimately the properties of the material change [39,40]. To evaluate the potential impact of algal substrate (*Ascophyllum nodosum*) ionic content upon the final properties of the material, the concentration of key ions has been suggested to lie between 0.7 and 3.9 wt% of sodium, 1.68 and 3.9 wt% of chloride, 0.2 and 1.6 wt% of magnesium, 1.5 and 2.6 wt% of potassium, 0.8 and 2.6 wt% of calcium, and 0.04 and 0.45 wt% of manganese [41]. Of the total fraction, sodium content was below 3.9 wt%, which equates to 1.56 g sodium per 40 g/L of *Ascophyllum nodosum* employed. This fraction is unlikely to significantly affect the productivity or the type of seaweed seeing as within the tested dependent conditions, upwards of 50 g/L of NaCl was tested. Differently, it is possible that the combination of potassium, magnesium, and calcium could result in their association with some of the γ-PGA synthesised.

**Figure 2:**
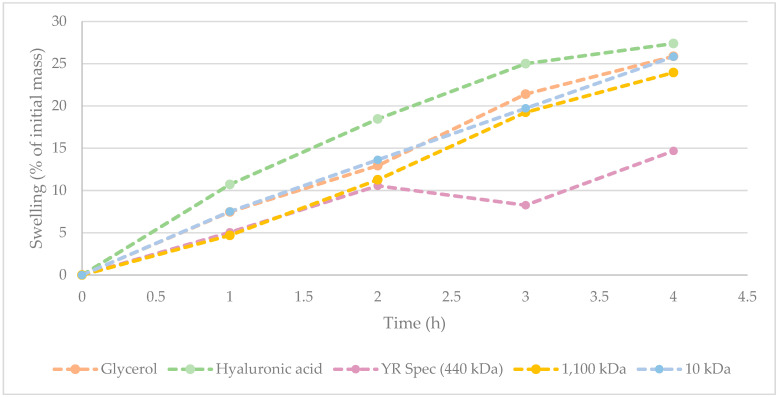
Hygroscopicity of commercially available γ-PGA against glycerol and commercially available hyaluronic acid. All values were normalised against initial weights of starting material following drying.

Hydration/humectant potential of γ-PGA [25,26,34], and more specifically, algal-derived γ-PGA, is key to assessing its ability to displace hyaluronic acid and other synthetic polymers. To this end, the hydration/humectant capacity of three commercially available γ-PGA samples was recorded according to the method described within Section 2.5. The values obtained from γ-PGA were compared to hyaluronic acid and glycerol, with widely recognised high hygroscopicity. The results from this investigation are summarised within Figure 2.

Figure 2 suggests that hyaluronic acid displays the greatest hygroscopicity across from 0 to 4 h. Interestingly, hygroscopic behaviour of 10 kDa commercial sample of γ-PGA was comparable to that of glycerol. At t = 4 h, there was only a marginal difference between water absorbed by hyaluronic acid, glycerol, and 10 kDa commercial sample of γ-PGA. Supporting the data observed within Table 6, it appears that variation in water absorbance between 10 kDa and 1100 kDa commercial samples of γ-PGA from the same supplier was marginal. Interestingly, the hygroscopicity displayed by YR Spec commercial γ-PGA was significantly lower compared to other samples at t = 3 h and 4 h, suggesting that the amorphous properties of this material might be less suitable for prolonged hygroscopic behaviour.

**Figure 3:**
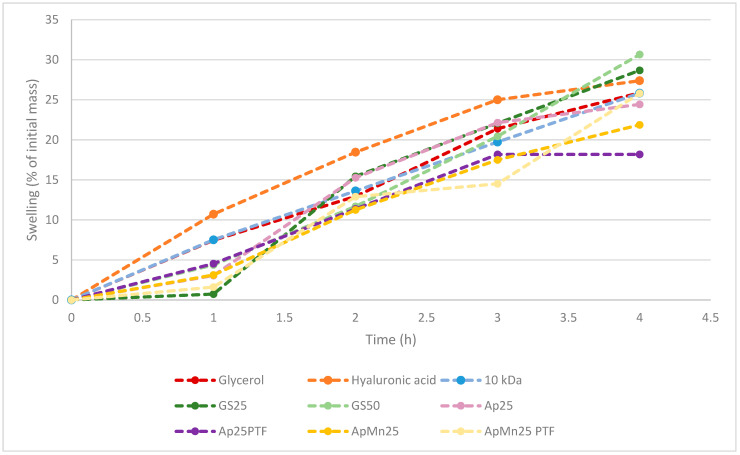
Comparison between hygroscopicity of benchmark molecules (hyaluronic acid, glycerol, and 10 kDa commercial γ-PGA) against γ-PGA produced from standard or algal media by *Bacillus subtilis* natto. All samples had not undergone additional purification through tangential flow filtration unless specified. All values were normalised against initial weights of starting material following drying.

In line with the previous observation, it is evident with both standard and algal substrates that the higher crystallinity within the structure is more favourable for interaction with water compared to amorphous structures. In fact, GS25, ApMn25PTF, Ap25, Ap25PTF, and ApMn25 all displayed lower initial tendencies to absorb water compared to GS50 samples. Depending on the sample, the discrepancy between crystalline and amorphous hygroscopicity was somewhat reduced over time. Notably, it appears that the t = 4 h total hygroscopicity was significantly different between standard media GS25 and *Ascophyllum nodosum*-derived media. From the data displayed within Figure 3, it can be hypothesised that initial hygroscopicity was largely dictated by material crystallinity, whereas as time passed, hygroscopicity was further affected by polymeric molecular weight. Notably, hygroscopicity recorded at t = 4 h for both GS25 and GS50 was higher compared to that of hyaluronic acid. The data obtained for ApMn25PTF suggest only marginally lower water absorption at t = 4 h compared to hyaluronic acid.

**Figure 4:**
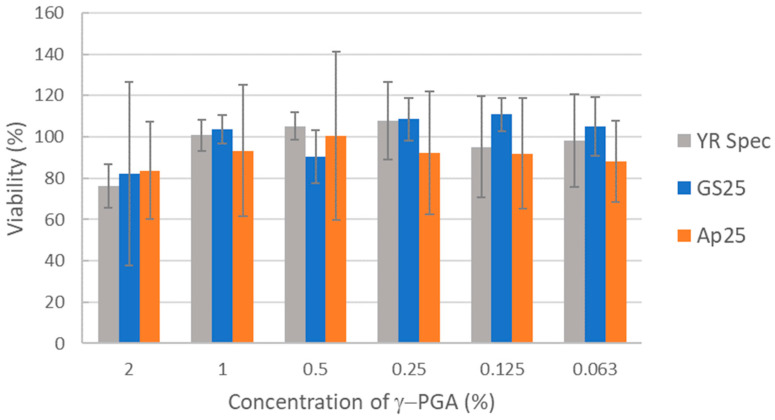
Comparison of biocompatibility of γ-PGA prepared from GS25 and Ap25 media to commercial γ-PGA. Human HaCaT keratinocytes were incubated with γ-PGA at the concentrations indicated for three days before viability was measured via XTT assay. Average values from three independent biological replicates are shown. Error bars represent the standard deviation.

Biocompatibility of γ-PGA samples were tested in the widely employed HaCaT human keratinocyte model. HaCaT cells were incubated with γ-PGA samples dissolved in medium at a range of concentrations from 2% downwards. Percentage viability was then measured by the XTT colorimetric assay based on metabolic activity. γ-PGA produced from standard medium (GS25) and *A. nodosum*-derived medium (Ap25) both showed comparable biocompatibility to the commercial YR Spec standard γ-PGA. For all γ-PGA samples tested, some effects on viability were observed at the highest concentration tested (2%), but these were relatively minor. Some of these minor effects could be a result of physical accumulation of material on the cell rather than detrimental biochemical interaction between γ-PGA and the cell (Figure 4).

**Figure 5:**
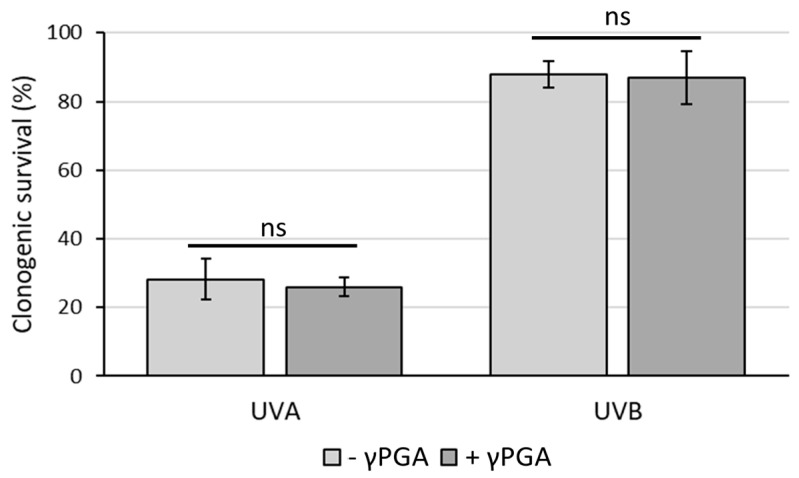
Clonogenic survival of HaCaT keratinocytes treated with UVA (50 kJ/m^2^) or UVB (800 J/m^2^) in the presence or absence of 1% γ-PGA. Survival is plotted relative to the plating efficiency seen for the respective unirradiated cells. Average values from three independent biological replicates are shown. Error bars represent the standard deviation. Analysis of the statistical significance was carried out by unpaired *t*-test. ns—not significant.

UV protection is another key factor within the cosmetics industry [42]. γ-PGA has not yet been reported to have either UVA or UVB photoprotective properties or photosensitisation [43] properties. For this reason, the clonogenic survival assay was used to test whether γ-PGA could affect killing of HaCaT keratinocytes by UVA or UVB. It was observed that incubation of cells in medium containing commercial γ-PGA (YR Spec) at 1% did not lead to either photoprotection against or photosensitisation towards either UVA (50 kJ/m^2^) or UVB (800 J/m^2^) (Figure 5). Although this suggests that γ-PGA is safe for inclusion in skin creams, it did not confirm the ability of these particular commercial grades to improve survival rates of keratinocytes to dangerous UV rays. Photoprotective mechanisms against UV by chemical agents can occur either by physically absorbing harmful photons [44] or through biochemically counteracting damaging processes (e.g., through antioxidant activity) [45]. These data suggest that commercial γ-PGA with 440 kDa when incorporated in cell culture medium at the concentration tested does not provide either biochemical protection or a sufficient physical barrier to prevent harmful effects of UV rays. 

**Figure 6:**
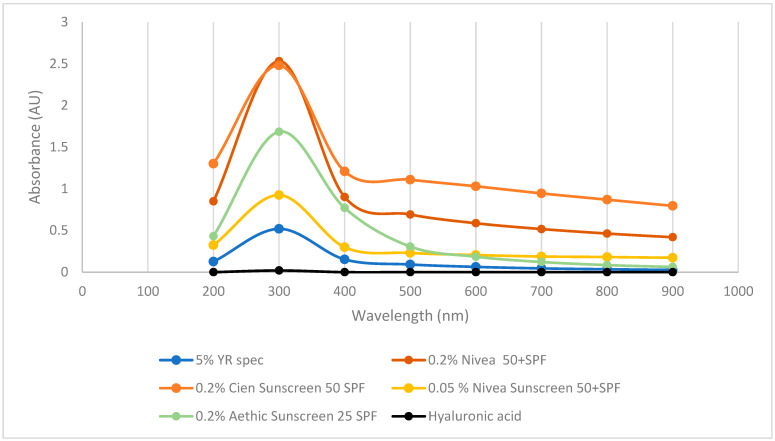
Absorbance of commercial sunscreen vs. hyaluronic acid vs. commercially available γ-PGA.

To further investigate whether γ-PGA has photoprotective properties, its UV-VIS spectrum was compared to hyaluronic acid and a range of suncreams. As previously reported in the literature, commercial hyaluronic acid did not display any absorbance across the tested wavelengths. Differently, three commercially available sunscreens displayed strong absorbance at 300 and 400 nm key ranges for blocking UVA and UVB (Figure 6). Interestingly, commercially available YR Spec γ-PGA did show significant absorption at 300 nm and some absorption at 400 and 500 nm; however, the concentration of this component was 10 times higher compared to commercially available sunblock.

**Figure 7:**
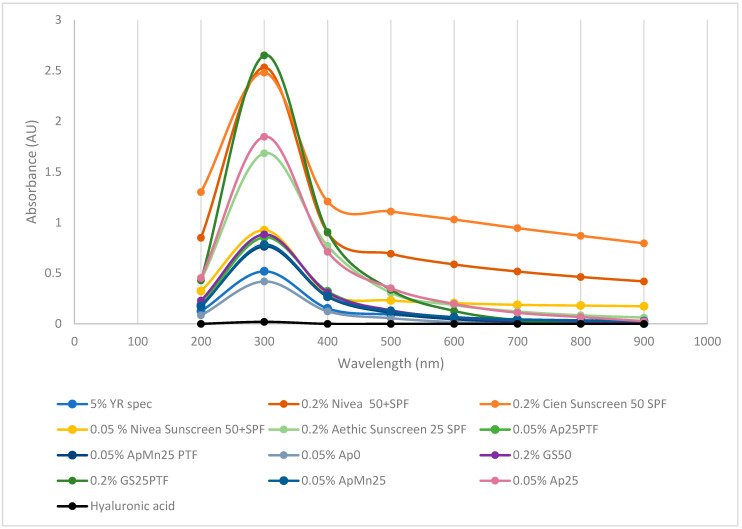
Absorbance of defined media γ-PGA vs. algal media γ-PGA vs. commercial γ-PGA vs. commercial sunscreen vs. marine safe commercial sunscreen vs. commercial hyaluronic acid.

The potential efficacy of defined media and complex algal media γ-PGA as sunblock was further assessed; the results are summarised within Figure 7.

Surprisingly, the produced γ-PGA, when dissolved at 0.2 or 0.05% concentrations, displayed significantly higher absorbance (300, 400 nm) compared to commercially available γ-PGA at 5%. Interestingly, at analogous concentration (0.2%), GS25PTF displayed higher absorbance at 300 nm compared to commercial Nivea 50+ SPF, Cien 50 SPF, and Aethic 25 SPF. Differently from hygroscopicity, it appears that UV absorption is more effective through amorphous material (GS25PTF, AP25PTF, ApMn25, AP25) compared to crystalline material (GS50, YR spec). Such observations are particularly surprising as, in the case of solid lipid nanoparticles, most of the skin protection capacity is a direct result of its crystalline structure [46]. However, it has been described by [47] that a superior UV protection can be achieved when an active is uniformly present within an amorphous polymeric core. This does suggest that part of the observed UV protection can be conferred by small actives associated physically with γ-PGA and not the γ-PGA itself. In fact, it has been widely reported that extracts from both brown and red seaweed do present strong UV protection properties, thanks to the abundance in microsporine-like amino acids [46,48]. Ap0 material also did not display particularly good UVA absorption despite being amorphous. It is possible that significant amounts of algal contaminants are present within the precipitate solution, and therefore these could impede on the three-dimensional distribution of the γ-PGA chains and ultimately on the capacity of this sample to absorb UVA.

**Figure 8:**
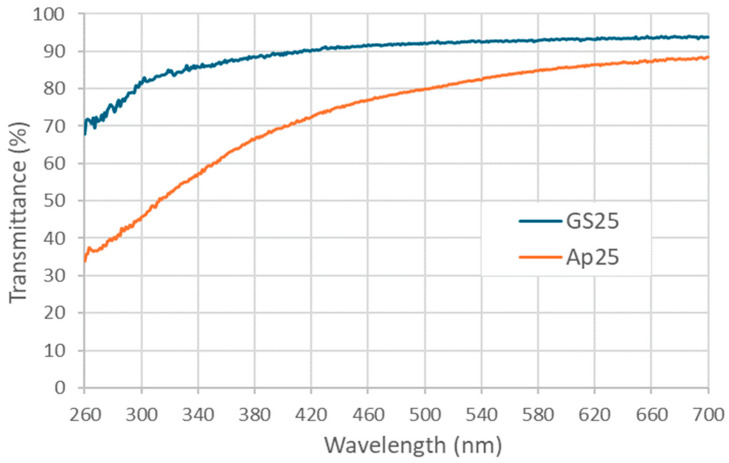
Transmittance spectrum of creams prepared with GS25 and Ap25 γ-PGA samples when applied to fused quartz plates at a density of 2.5 mg/cm^2^.

Further, to the observed UV absorbing properties of γ-PGA (Figure 6 and Figure 7), spectroradiometry was used to investigate the potential UV screening effects of two creams formulated with GS25 and Ap25 γ-PGA, separately (Figure 8). Both creams showed good potential for UV protection, decreasing transmittance most significantly in the sub-400 nm region. Notably, and consistent with the data shown in Figure 7, the cream prepared using *A. nodosum*-derived medium showed more promising optical properties than the cream containing γ-PGA prepared from GS25 medium, screening out half or more of the radiation at wavelengths in the UVB range when applied at 2.5 mg/cm^2^ (Figure 8). For example, the T_315_ for GS25 γ-PGA cream was 84%, while for the Ap25cream, it was 51%. Provided that the molecular weight of GS25 was significantly higher than that of Ap25, a reasonable explanation for the reduction in transmittance is that non-γ-PGA components of Ap25 are responsible for the screening effect. This is reasonable, as γ-PGA could be strongly interacting with some algal compounds, which are not fully removed during purification.

**Figure 9:**
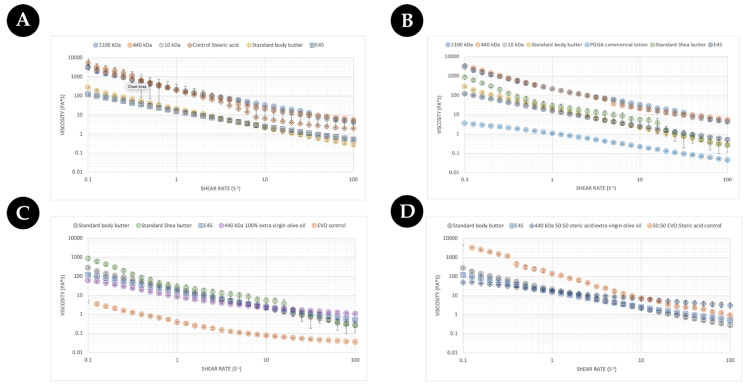
(**A**) Comparison of a commercial butter against commercial γ-PGA (with molecular weights of 1100 kDa, 440 kDa, and 10 kDa) creams formulated with 100% steric acid as per Table 1 (**B**) Comparison of more commercial butters/hydrating creams against commercial γ-PGA (with molecular weights of 1100 kDa, 440 kDa, and 10 kDa) creams formulated with 100% steric acid as per Table 1. (**C**) Comparison of commercial butters/hydrating cream against a γ-PGA cream formulated with 100% extra virgin olive oil as per Table 1. (**D**) Comparison of commercial butters/hydrating cream against a γ-PGA cream formulated with 50:50 extra virgin olive oil/steric acid as per Table 1. Data represent the average of triplicate measurements. Error bars summarise the standard deviation of the formulations.

From a consumer’s point of view, the feeling of the cream/lotion/moisturiser constitutes a key part of the purchasing decision. To this end, the rheological properties of different commercial γ-PGA were assessed against an array of commercially available creams, body butters, and lotions to evaluate potential excipient integration/substitution based upon the final desired formulation. Initially, the three types of commercially available γ-PGA types were formulated with steric acid as a base material and compared to standard body butter and E45 cream. The data are summarised within (Figure 9). As expected, the flow profile of the standard body butter and the E45 cream were very similar. As was further expected, all tested creams showed shear thinning behaviour as the viscosity decreased with the increased shear rate [49]. Overall, the rate at which the viscosity decreased was greater for creams when compared to the lotion (Figure 9B) and Evo control (Figure 9C), which are less sensitive to the change in shear rate. Surprisingly the flow behaviour of steric-acid-based commercial γ-PGA samples was comparable to that of the controls but significantly different to the flow profile of commercial creams. Interestingly the behaviour of steric acid was similar to that of γ-PGA creams at shear rates below 10 (s^−1^), after which the viscosity decreased significantly from 5000 (Pa*s) to approximately 9 (Pa*s) at 100 (s^−1^). The viscosity at about 100 (s^−1^) of shear rate has been associated with the ease of rubbing a cream [50], and lower viscosity at a higher shear rate suggests a better spreadability upon topical application. These data suggest that the main component of the cream is significantly more viscous compared to commercial creams and that γ-PGA controls maintain the viscosity significantly higher at higher shear rates compared to the steric acid control, which would have a better spreadability.

Regardless, to better understand the variation between γ-PGA-based formulations and more cosmetic formats, the flow profile was further compared with a commercial γ-PGA-based lotion sold by Boots, UK, and another commercially available butter (Figure 9B). The γ-PGA lotion displayed approximately Log 4 less viscosity across all shear rates compared to steric-acid-based γ-PGA formulations. Differently, the commercially available shea butter presented a rheological profile in between that of steric-acid-based γ-PGA, standard body butter, and E45.

In an attempt to further elucidate the effect of γ-PGA on the rheological of creams with different base lipids, the steric acid component was completely replaced with extra virgin olive oil. For comparison purposes, only one of the three commercial γ-PGAs was employed to formulate this new cream formulation as no significant variation in rheological profile between different commercial γ-PGAs with the same base materials were observed (Figure 9A,B). It has been previously described within the literature the beneficial effect of extra virgin olive oil (EVO) on the skin [51]. The effect of extra virgin γ-PGA as a rheological modifier to improve the sensation of the cream on the skin was further investigate (Figure 9C). It can be observed from Figure 9C that at lower shear rates, EVO-based formulations presented lower viscosity compared to E45 and body creams; differently, at higher shear rates, the viscosity became comparable or higher compared to both E45 and body creams, suggesting shear-dependent thickening commonly observed with gums, cyclodextrin, and the resulting interaction of the two species [52,53,54]. From our observations in Figure 9A–C, if the consistency of creams or butters is required, it could be further improved by substituting the lipid component (Figure 9C) or by tailoring the ratio between more viscous steric-acid-like components and less viscous oily components such as EVO or similar (Figure 9D).

To further validate the hypothesis on the ratio of steric acid and EVO and the resulting flow profile, a 50:50 EVO/steric acid formulation was assessed within Figure 9D. Surprisingly, the variation in rheological profile between an EVO-only formulation and a 50:50 EVO/steric acid formulation was not as expected. Firstly, the initial viscosity of the cream was not significantly different between the two. In fact, the variation was observed in the delta viscosity between low and high shear rates, where, with the EVO-only formulation, this was quite significant and, differently from the 50:50 EVO/steric acid formulation, the viscosity was maintained quite constantly throughout the shear spectrum. This was particularly surprising as it was the opposite of what was observed with the EVO-only rheological profile (see Figure 9C) in which the delta viscosity was lower across the shear spectrum compared to the higher delta viscosity for the 50:50 EVO/steric acid mixture, as displayed in Figure 9D. Such variation in properties underlies the crucial effect that formulation components, including γ-PGA, exhibit on the overall formulation as a function of shear rates. It is therefore inherently more complex to create formulations which such active components compared to smaller-molecule-dominant formulations.

Further to investigating the impact of the ratio between more viscous and less viscous components, the effect of different lipids was evaluated. For instance, within Figure 9D, we summarise the effect of beeswax on the overall cream formulation compared to beeswax- and EVO-based formulations. Interestingly, beeswax displayed even lower delta viscosity as a function of shear rate throughout the spectra. It will be therefore crucial to further investigate the behaviour of intra-formulation (gum Arabic, cyclodextrin, γ-PGA) with the lipid component and their effect upon the rheological profile of the cream.

## 4. Conclusions

Further from the successful report by Parati et al. [24] on the capacity to synthesise γ-PGA from whole seaweed, we herein report that *Bacillus subtilis* natto, cultivated on whole brown seaweed, is affected by the concentration of Mn^2+^. This effect was previously described in defined media for *B. subtilis*; however, not for any complex media. This preliminary investigation was able to confirm the ability to tune the chemical properties of γ-PGA by means of auxiliary fermentation elements (Mn^2+^), with a significant effect on both the hygroscopicity and UV protection effect of the resulting γ-PGA. Further, our data presented a lack in rheology variation between commercial γ-PGA-based cream, which was solely driven by the lack of accurate and comprehensive material characterisation, strongly emphasising the lack of reliable commercial γ-PGA material. Regardless, our data suggest the ability to formulate both commercial and seaweed-derived γ-PGA within a model cream system in order to achieve a stable product. Further, MTT assay confirmed that γ-PGA synthesised by both GS and seaweed-derived media does not detrimentally affect the viability of HaCaT keratinocytes.

Although a strong preliminary investigation was undertaken, to develop the next generation of cosmetic products, critical consideration on the persistence of the cream on the skin, impact of the cream components on the skin microbiota, antioxidant effect of the formulation components, and optimisation of the UV protective characteristics of the formulation would all be crucial parameters to better pinpoint the synergy (or lack thereof) between skin and product, with a positive effect over the natural ecosystem (i.e., impact on ocean ecosystem), anthropogenic wellbeing, and longevity.

## Figures and Tables

**Table 1:** Defined and complex medium composition for the biosynthesis of γ-PGA by *Bacillus subtilis* natto.

Medium Name	Components	Quantity (g/L)
GS	Sucrose	50
	L-Glutamate	20
	NaCl	50
	KH_2_PO_4_	2.7
	Na_2_HPO_4_	4.2
	MgSO_4_·7H_2_O	5
	Murashige–Skoog vitamin solution	1 mL/L
GS25	Sucrose	50
	L-Glutamate	20
	NaCl	25
	KH_2_PO_4_	2.7
	Na_2_HPO_4_	4.2
	MgSO_4_·7H_2_O	5
	Murashige–Skoog vitamin solution	1 mL/L
GS0	Sucrose	50
	L-Glutamate	20
	NaCl	0
	KH_2_PO_4_	2.7
	Na_2_HPO_4_	4.2
	MgSO_4_·7H_2_O	5
	Murashige–Skoog vitamin solution	1 mL/L
Ap0	*Ascophyllum nodosum*	40
	Sucrose	25
	L-Glutamate	10
	NaCl	0
Ap25	*Ascophyllum nodosum*	40
	Sucrose	25
	L-Glutamate	10
	NaCl	25
Ap50	*Ascophyllum nodosum*	40
	Sucrose	25
	L-Glutamate	10
	NaCl	50
ApMn0	*Ascophyllum nodosum*	40
	Sucrose	25
	L-glutamate	10
	NaCl	0
	MnSO_4_	0.415 g/L
ApMn25	*Ascophyllum nodosum*	40
	Sucrose	25
	L-Glutamate	10
	NaCl	25
	MnSO_4_	0.415 g/L
ApMn50	*Ascophyllum nodosum*	40
	Sucrose	25
	L-Glutamate	10
	NaCl	50
	MnSO_4_	0.415 g/L

**Table 2:** Standards employed for hygroscopicity testing.

Commercial Name	Supplier	Type
440 kDa	YR Spec	Commercial γ-PGA
10,000 Da	Bonding Chemical	Commercial γ-PGA
1100 kDa	Bonding Chemical	Commercial γ-PGA
Hyaluronic acid 1000 kDa	Peak Supplements (Bridgend, UK)	Commercial bacterial hyaluronic acid
Glycerol	Special Ingredients	Hygroscopic plant-based sugar

**Table 3:** Commercial creams employed as rheological standards.

Commercial Name	Supplier	Type
E45	Boots	Moisturising cream
Shea Body Butter	The Body Shop	Moisturising butter
Meringa Body Butter	The Body Shop	Moisturising butter
γ-PGA Lotion	Boots	Hand lotion
SPF 50+ Nivea	Nivea	UV protection cream
Cien SPF 50+		Sunscreen
SPF 25+	Aethic	Sunscreen marine safe

**Table 4:** Components and amounts (in %) of γ-PGA cream formulation. If otherwise not specified throughout the manuscript, 100% of the fat content is stearic acid.

	10 kDa	440 kDa	1100 kDa
γ-PGA	2	2	2
Gum Arabic	2	2	2
Potassium hydroxide	0.25	0.25	0.25
Stearic acid	6	6	6
Extra virgin olive oil	0	0	0
Cyclodextrin	2	2	2
Distilled water	16	16	16
Essential oil (lemongrass oil)	100 μL	100 μL	100 μL

**Table 5:** Cream formulations prepared and their pH values.

Cream Formulation	Description	pH
440 kDa	Lotion cream with 440 kDa γ-PGA	7.05
10 kDa	Lotion cream with 10 kDa γ-PGA	7.11
1100 kDa	Lotion cream with 1100 kDa γ-PGA	6.93

**Table 6:** Physical characteristics of commercial γ-PGA and γ-PGA synthesised from GS/algal media. Samples marked with * are aggregated and *** are highly aggregated; as such, the calculation was carried out only for the range where there was good separation on the columns. The displayed data underwent tangential flow filtration purification through a 30 kDa membrane.

Production Method	M_n_ [kDa]	M_w_ [kDa]	M_w_/M_n_	XRD
YR spec γ-PGA	250	440	1.8	Amorphous
Bonding chemical 10,000	115	216	1.9	Semi-crystalline
Bonding chemical 1,100,000	120	250	2.1	Semi-crystalline
GS, 0 g/L NaCl PTF [24]	3320	3700	1.1	Amorphous
GS, 25 g/L NaCl PTF [24]	1900	2310	1.2	Semi-crystalline
GS, 50 g/L NaCl PTF [24]	1810	2700	1.5	Crystalline
GS, 25 g/L NaCl + MnSO_4_ PTF [24]	740	1280	1.7	Crystalline
Ap, 0 g/L NaCl PTF ^(^*^)^	66	210	3.2	Amorphous
Ap, 25 g/L NaCl PTF ^(^*^)^	160	250	2.8	Amorphous
	13	36	1.6	
	12	24	2.0	
Ap, 50 g/L NaCl PTF ^(^*^)^	38	102	2.7	Amorphous
ApMn, 0 g/L NaCl PTF ^(^***^)^	250	900	3.6	Amorphous
ApMn, 25 g/L NaCl PTF ^(^*^)^	62	152	2.5	Amorphous
ApMn, 50 g/L NaCl PTF ^(^*^)^	66	150	2.3	Amorphous

## Data Availability

Data are contained within the article.
